# Excision of an Ulcerated Scrotal Hemangioma in a Four-Month-Old Boy: A Case Report and Review of Literature

**DOI:** 10.7759/cureus.25844

**Published:** 2022-06-11

**Authors:** Wael M Moneir, Ahmed AlShammari

**Affiliations:** 1 Department of Pediatric Urology, King Abdullah Specialized Children's Hospital, King Abdulaziz Medical City, Ministry of National Guard Health Affairs, Riyadh, SAU

**Keywords:** surgical excision, genital ulcer, scrotal ulcer, ulcerated hemangioma, infantile hemangioma

## Abstract

Although hemangioma is a common benign tumor of childhood, hemangioma of the genitalia is rarely seen. Multiple treatment options are available for the management of ulcerated hemangiomas but deciding on surgery to treat an ulcerated genital hemangioma is usually difficult due to the lack of guidelines addressing this entity. Here we report a case of an ulcerated hemangioma of the scrotum, that was managed by surgical excision in a four-month-old boy, and a review of related literature.

## Introduction

Infantile hemangioma (IH) is the most common tumor of childhood. IHs in certain locations on the skin can be associated with unique medical concerns [1]. Ulcerated scrotal hemangiomas are rare tumors, and evidence regarding optimal management is lacking. In this report, we describe the surgical management of an ulcerated scrotal hemangioma, and the rationale for the selected treatment. The present work has been reported in accordance with the Surgical Case Reports (SCARE) criteria [2].

## Case presentation

A four-month-old male patient was presented to our emergency department pursuing further management for an ulcerated lesion over an already known hemangioma on the anterior surface of the right hemi-scrotum. The patient was a full-term boy with negative antenatal history and a normal spontaneous vaginal delivery.

The patient was initially managed in another healthcare facility; his hemangioma was diagnosed soon after birth, and a pediatrician followed its natural history of uncomplicated rapid growth. However, two months before his presentation, the hemangioma started to ulcerate without discharge, and it was associated with discomfort and mild pain during urination and defecation.

**Figure 1:**
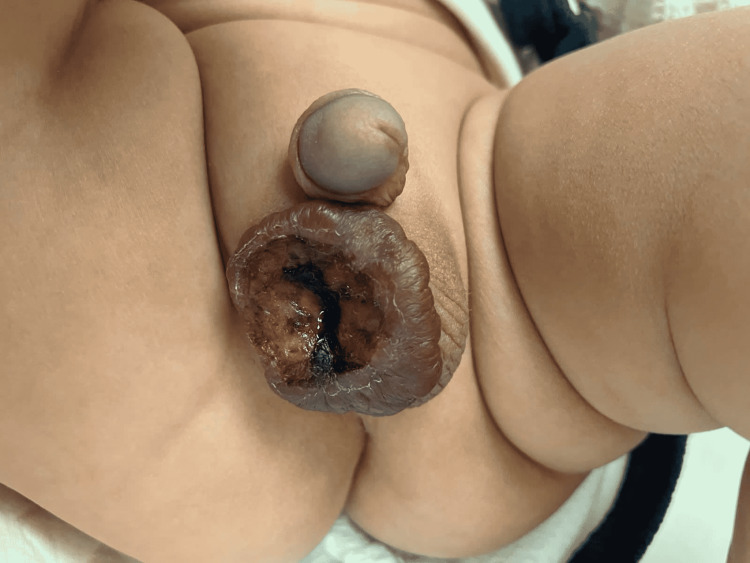
Scrotal hemangioma on presentation

General examination was unremarkable and the baseline blood investigations were within normal range. Local physical examination showed a normal circumcised penis and a hemangioma measuring 4 x 4.5 cm, occupying the anterior surface of the right hemi-scrotum totally. Moreover, extensive ulceration covering almost the entire hemangioma surface was also observed. The ulcer was 5 mm deep, indurated with punched-out edges. The floor of the ulcer was a mixture of granulation tissue and slough. No bleeding or discharge was noted (Figure 1). The hemangioma was extending deep into the scrotum smoothly. The right testis was palpable in the groin. The left hemi-scrotum and the left testis were normal during the examination.

**Figure 2:**
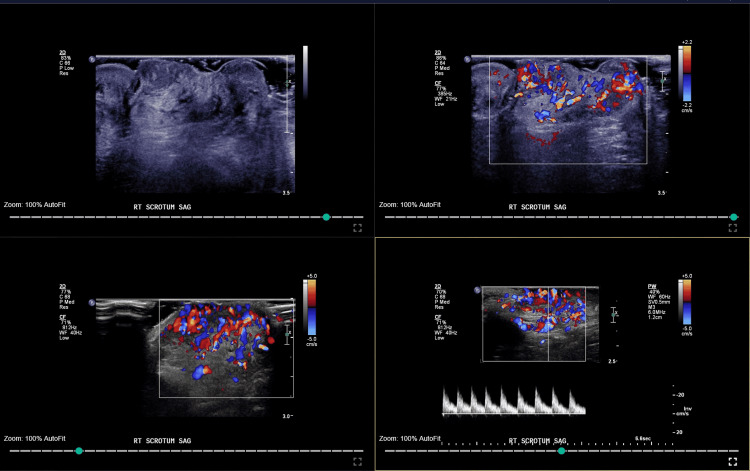
Doppler ultrasonography scan of the scrotum showing the heterogeneous hypervascular lesion

A color Doppler ultrasonography scan of the scrotum showed that the right scrotum was occupied by a heterogeneous hypervascular lesion (Figure 2). A normal left testis within the scrotum and a normal right testis within the inguinal canal with normal epididymis were seen bilaterally.

A pediatric urologist, a dermatologist, and a general pediatrician assessed the patient. Possible management options including medical treatment and surgical excision were explained to the parents. After a discussion with the parents, a decision for surgical excision was made.

**Figure 3:**
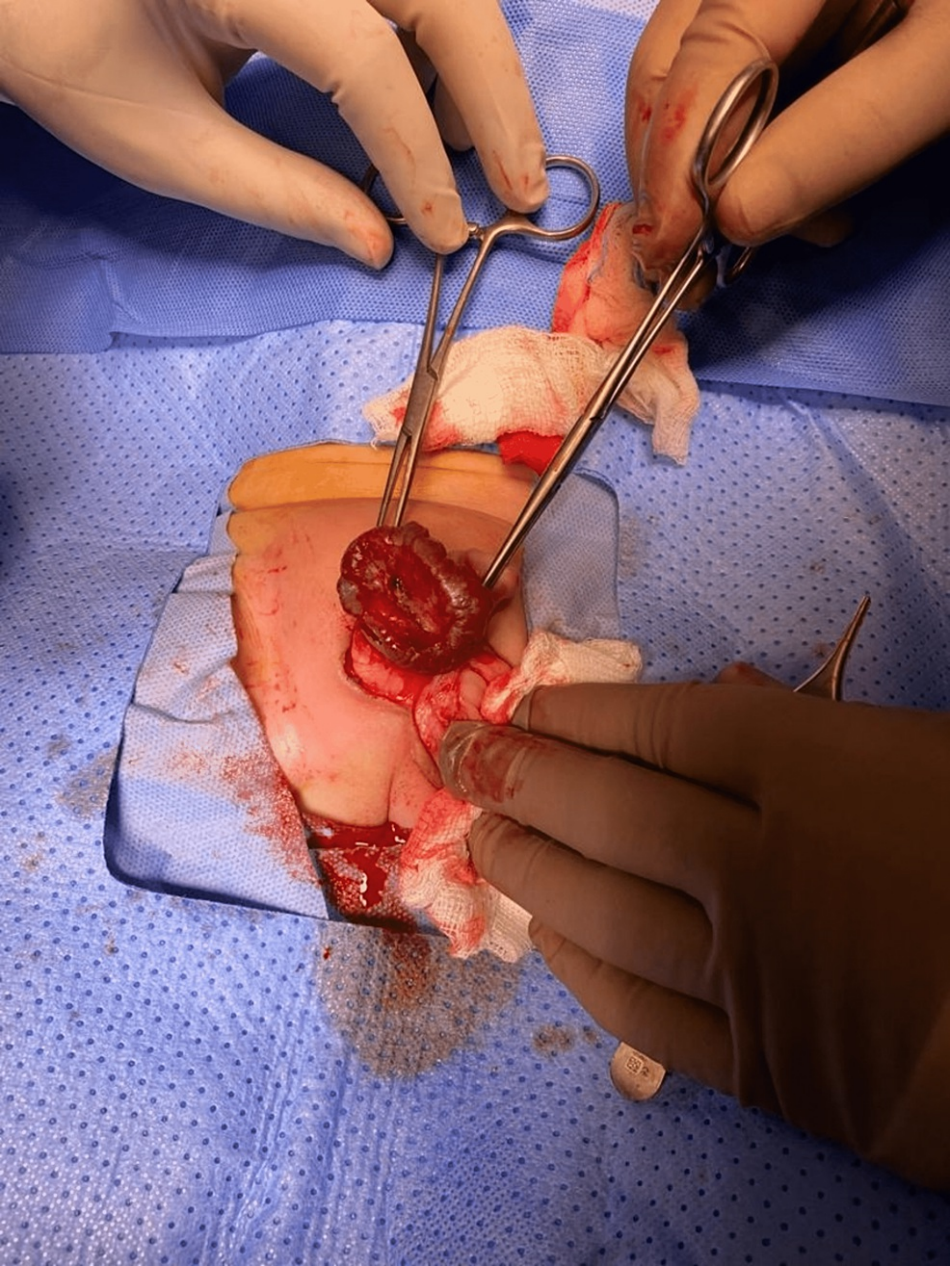
Dissecting the hemangioma

**Figure 4:**
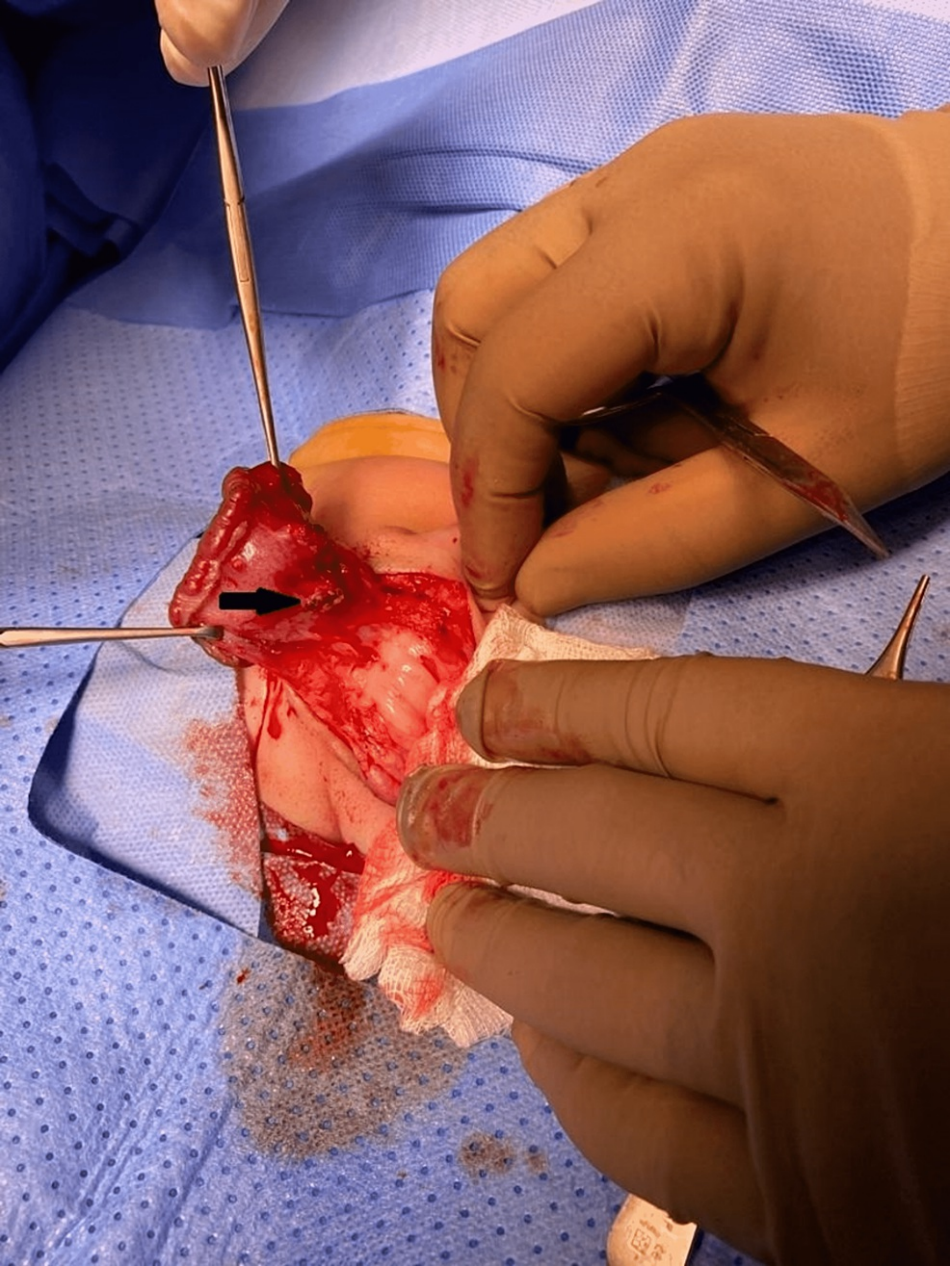
Identifying and ligating the feeding vessel (arrow)

**Figure 5:**
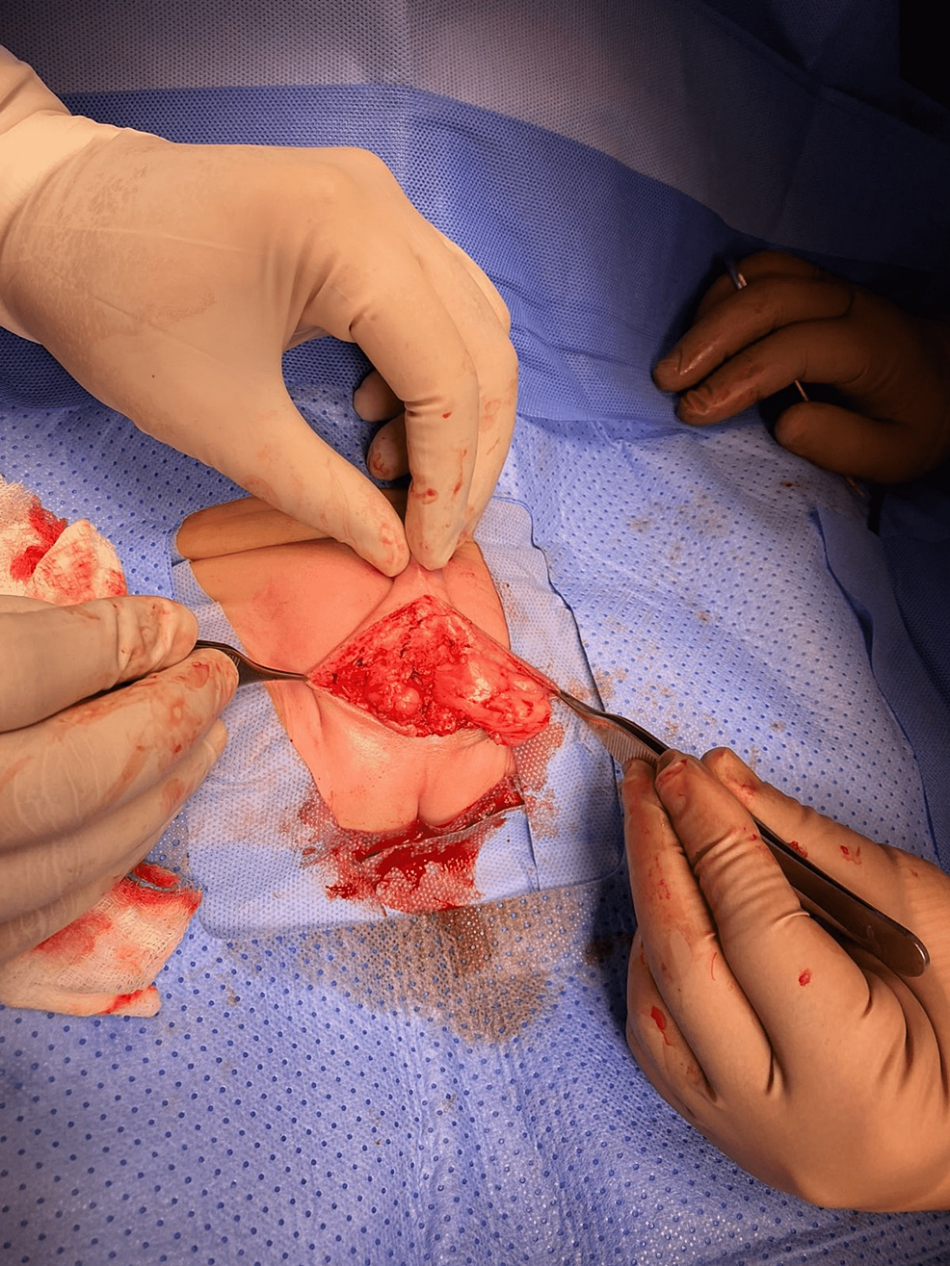
Closure of the defect after the excision of the hemangioma

Under general anesthesia and caudal block, skin limits of the lesion were marked. A circumferential and tapered incision on healthy scrotal skin surrounding the lesion was made, followed by progressive dissection of the scrotal layers; the pedicle of the feeding vessel was identified and ligated. The hemangioma was entirely excised and the specimen was sent for histopathologic examination (Figures 3-5).

**Figure 6:**
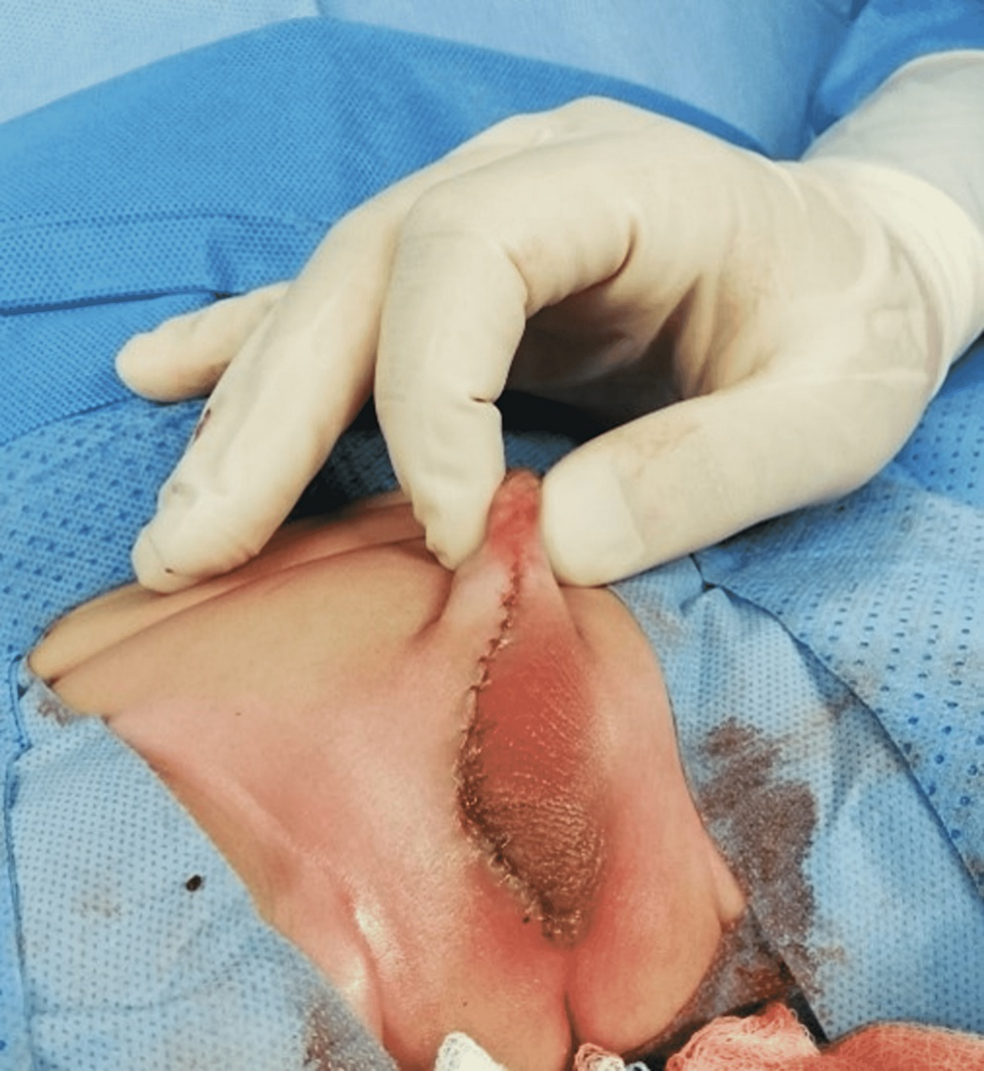
Immediate postoperative view

We were able to restore the scrotal defect that remained after the excision of the hemangioma with primary closure (Figure 6).

The patient, who tolerated the procedure well, was admitted for postoperative observation. He was discharged home in a good condition, 24-hours after the surgery. The histopathology exam confirmed the diagnosis of IH.

Six weeks postoperatively, the patient was brought for a follow-up visit. He was doing fine with healed wounds and no complaints. Right orchidopexy after a six-month interval was planned with regular periodic clinic visits.

## Discussion

IH is the most common benign tumor in infancy with an estimated incidence of 5% [1]. They vary enormously from small, harmless, self-resolving, requiring no treatment to large, potentially problematic, and function-threatening tumors. Hemangiomas have a distinctive natural history and specific growth characteristics. Most hemangiomas arise in the first few weeks to months of life and pass through a rapid growth phase during infancy followed by involution during the first few years of life [1,3-6].

Diagnosis of IHs usually depends on physical findings. Routine imaging is not recommended for localized hemangioma. However, when the diagnosis is uncertain, or there is a concern of associated structural abnormalities, magnetic resonance imaging (MRI) and ultrasound evaluation may be considered [1,3,4,6,7].

Hemangiomas can be classified based on their depth (superficial, deep, and combined), anatomical pattern (focal, segmental, and multifocal) [5], or risk stratification (high risk and low risk). The latter classification has implications for the timely identification of hemangiomas that may require early intervention [1,3,4,6].

High-risk lesions include hemangiomas that may cause permanent scarring and disfigurement, hepatic or airway hemangiomas, and hemangiomas with the potential for functional impairment, ulceration, or associated underlying abnormalities. These hemangiomas should prompt concern, emphasizing increased attention and consideration of early treatment [1,3-6]. Ulceration is a common complication of IHs and can result in significant morbidities. Complications of ulceration included pain, bleeding, interference with daily activities, infections, functional impairment, and scarring; the diaper area had a higher risk of these complications [5,8,9]. Large scrotal hemangioma has its unique complication; it may be associated with testicular damage, with a possible impact on the spermatogenic capability of the testicles assumed to be due to the high temperature generated by the hemangioma [10,11].

Early treatment is indicated in approximately 10% of IH cases [6]. Early intervention is recommended for high-risk IHs. When treatment is indicated, propranolol is the first-line therapy. Treatment usually is considered for at least six months and often is maintained until 12 months of age. Topical timolol may be used to treat selected IHs [1,6]. Surgery and laser treatment are usually reserved for the treatment of residual skin changes and scars after involution. Surgical excision may less commonly be considered earlier to treat some selected hemangiomas that are not responding to medical therapy, if there is a treatment contraindication, or when there is concern about compromise in the function of a vital organ [1,5].

Ulceration is usually an indication of intervention. Currently, available treatment options include wound care, medical therapy, laser therapy, and surgery [9]. However, a recent report with a large cohort has found that, although infants benefit from propranolol therapy in managing ulcerated IHs, many continue to experience prolonged healing times [8].

Large and ulcerated genital hemangiomas are quite a rare entity, and often pose diagnostic and treatment challenges for the treating surgeon. Various treatment options are described in the literature, but there is no clear consensus on its management due to its rarity [12,13]. Given the predictable course of IHs, a reported practice has been to observe such tumors unless they are causing functional risk, are symptomatic, or would impede time-sensitive reconstructive surgery in the future [14]. However, in cases of scrotal hemangiomas, scheduled surgical removal of the lesion, with preservation of anatomic structures of the scrotum is recommended [10].

In our case, no cross-sectional imaging investigation was recommended due to the localized nature of the lesion. Ultrasonography scan was used only to assess the characteristics of the undescended testis. For our patient, both conservative (wound care and propranolol) and surgical management were offered to the parents. The possible lengthy conservative management course, the possible need for future orchiopexy, and the concern of testicular damage were also explained. The decision for surgical excision was made following parental preference.

## Conclusions

Despite the presence of recent guidelines addressing the management of IHs, developing a rationale for interventions can be challenging. With the dearth of clinical evidence existing about the sequelae of ulcerated scrotal hemangioma, surgical excision is supported by the ambiguity about the effect of these lesions on testicular function and fertility. Until the capability to predict the prospective consequences of scrotal hemangioma is improved, surgical excision of these lesions could be a safe option. Furthermore, parental preferences, after a thorough discussion, might guide planning therapy and follow-up.
